# Social capital and refraining from medical care among elderly people in Japan

**DOI:** 10.1186/s12913-016-1599-8

**Published:** 2016-08-02

**Authors:** Masaaki Mizuochi

**Affiliations:** Faculty of Policy Studies, Nanzan University, 27 Seirei, Seto, Aichi Japan

**Keywords:** Refraining from medical care, Elderly, Social capital, Trust, Reciprocity, Community, Japan

## Abstract

**Background:**

Refraining from required medical care can worsen health, particularly for the elderly, and increase public medical expenditure, which destabilizes the financial aspect of social security. Social capital, such as trust between residents and the norms of reciprocity in the community, is a possible measure to prevent refraining from medical care.

**Methods:**

We studied survey data collected in a small area in Japan that included a high response rate (91.6 %) to evaluate refraining from medical care. Self-reported refraining from required medical care from among 1016 elderly people, aged ≥60 (male = 490; female = 526), was used as a dependent variable. Social capital indicators were mean values of people’s attitude toward the generalized trust and norms of reciprocity in each community. We estimated the association between community level social capital and individuals’ probability of refraining from medical care while controlling individual factors such as age, education, and marital status.

**Results:**

Logit estimation results showed that only generalized trust is associated with low probability of refraining from medical care among the elderly in small communities. The marginal effect for 0.1 increase in community level trust is 4 % decrease in the probability of refraining from medical care. In larger communities, generalized trust is not associated with the probability of refraining from medical care.

**Conclusions:**

This finding suggests that the generalized trust is effective in smaller communities as far as related to access to medical care. In small communities, policy to increase generalized trust to support medical care for elderly is recommended.

**Electronic supplementary material:**

The online version of this article (doi:10.1186/s12913-016-1599-8) contains supplementary material, which is available to authorized users.

## Background

The purpose of this paper was to examine the effect of social capital, trust, and norms of reciprocity in the community, with regards to refraining from medical care for elderly people in Japan. We also investigated whether the effects of this social capital differs by community size.

Access to medical care is a fundamental right and is an important determinant of health. Several studies have investigated the factors associated with refraining from medical care including economic conditions, educational attainment, employment conditions, and race [1–7]. In Japan, people with low income are more likely to refrain from medical care [8–10]. Previous studies in Japan have implicated other factors including marital status, socioeconomic class, and household income [11–15].

The population of Japan has begun to diminish because of a rapidly declining fertility rate; therefore, the number of small towns or communities is predicted to increase significantly in the near future. In Japan, the percentage of small municipalities with less than 5000 people has been projected to increase from 13.4 % (2010) to 22.0 % (2040) [16]. Moreover, the number of doctors per 10,000 people in a depopulated area is 13.75, which is comparatively lesser than the country’s average of 20.09 [17]. This means that smaller communities have more difficulty in accessing a doctor. As a result, people living in small towns have to go to a doctor in a distant area. Elderly people, in general, have difficulty in traveling due to physical or financial limitations. Elderly people who have no means of transportation participate less in health examinations [18]. Therefore, an increase in the number of individuals living in the small towns increases refraining from medical care among some elderly people. Clearly, this population trend can worsen the health status of the elderly and result in increased public medical expenditure. To prevent this scenario, cooperation within the community, such as provision of transportation and financial assistance, could help the elderly. However, public finance cannot afford to provide such assistance under the low growth rate economy. Therefore, we focused on the role of social capital, as explained below, in this cooperative activity.

Putnam defined social capital as “features of social organization such as networks, norms, and social trust that facilitate coordination and cooperation for mutual benefit” [19]. Social capital was also defined by other authors as the resources embedded within one’s social relationships and an individual attribute [20–22]. We focused on the ability of a community to encourage and help elderly people to go to a doctor. In this case, it was reasonable to consider social capital as a public good; that is, collective efficacy, as defined by Putnam. To investigate the effect of collective efficacy, we can use the indicators “generalized trust” and “norms of reciprocity” identified from the data used in this study.

We considered that two functions of social capital relating to health can be applied in public access to medical care [22]. First, if we help others in the same community, we can also expect help from others in case the need arises (enforceable trust). Second, people living in a high social capital community are likely to join a certain group and support elderly people seeking health care (appropriable social organization). There are also other mechanisms linking social capital to health care seeking [23, 24]. This collective efficacy can be generated by high levels of social capital to reduce the number of people refraining from medical care.

Effects of social capital on health have been confirmed by studies worldwide including Japan [25–32]. In some countries, the association between social capital and access to medical care has been studied; however, to the best of our knowledge, such studies have not been conducted in Japan [23]. The effect of social capital in the neighborhood on access to medical care has been found to be positive by many studies [33–37]; however, no association has been found between the two in a study [38]. Therefore, with the goal of improving the health condition of the Japanese population, we aimed to investigate this association in Japan, while taking into consideration the findings of the other studies in different countries. Here, we aimed to investigate the association between social capital and the probability of refraining from medical care in Japan.

Moreover, we examined the difference in its relation by community size. Considering the relationship between the size of the community and the effect of social capital, there were a few papers that revealed interesting findings. For example, in Sweden, the smaller the neighborhood, the bigger the contextual effect of poverty on mental disorder [39]. Moreover, in both small and large communities in Japan, social capital had a significant effect on preventing crime; although there was no effect in medium-sized communities [40].

## Methods

### Data source and survey area

The study survey was conducted by a research unit comprising staff from two faculties at Mie University in Japan, the Faculty of Medicine and Faculty of Humanity, Law and Economics. The research unit designed questionnaires by referencing existing questionnaires in Japan. The English language version of the questionnaire is presented in Additional file 1.

**Fig. 1:**
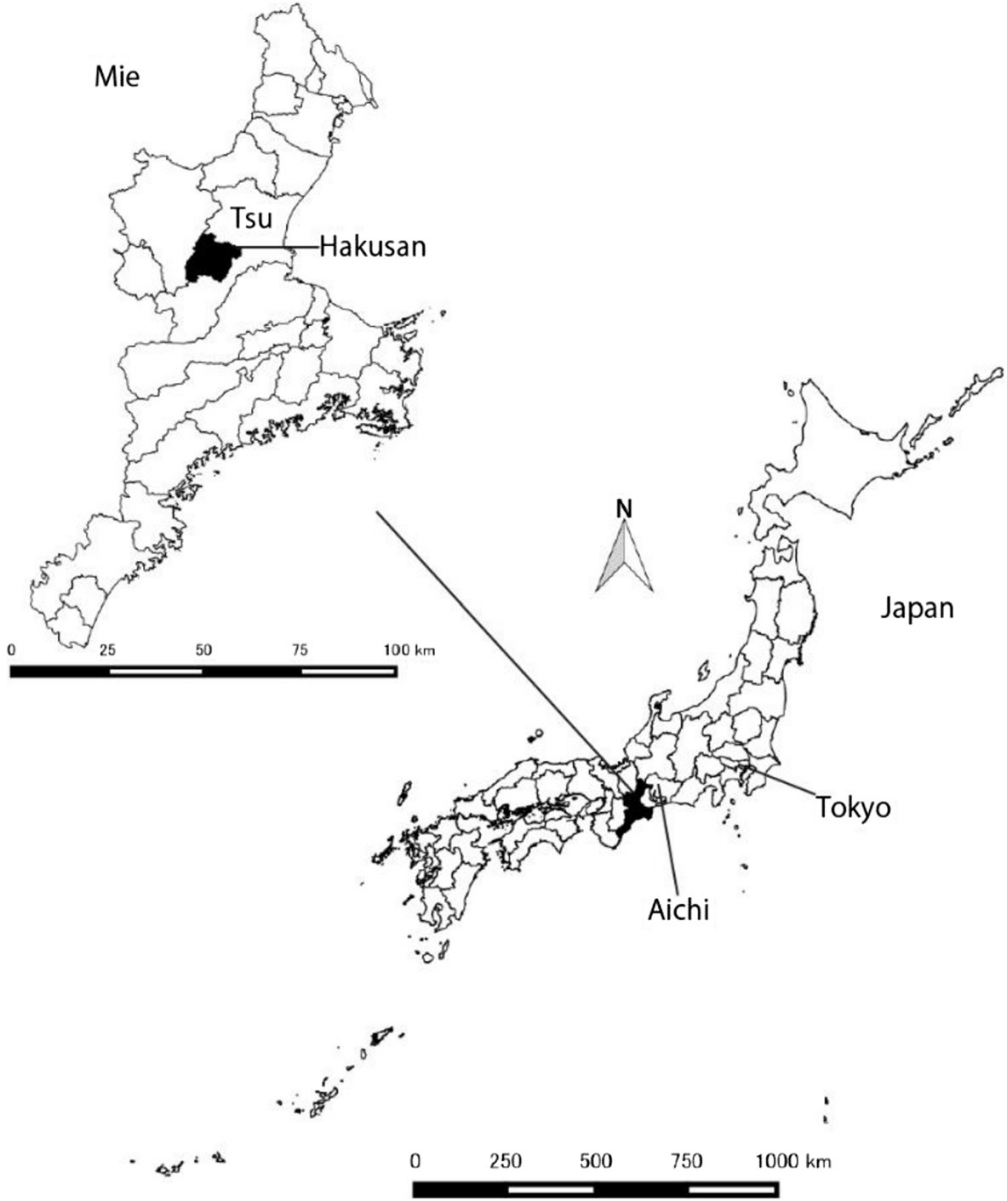
Map of the survey area

The survey area was Hakusan, a part of Tsu City in the Mie Prefecture in Japan (Fig. 1). The random sample studied comprised 3106 people (aged ≥20 years) living in the area during March 2012. Community officials visited respondents’ house to distribute questionnaires on September 1, 2012 that were collected by September 20, 2012. Respondents were unsupervised when completing questionnaires. The response rate was 91.6 % (*N* = 2844). The population of the Hakusan area aged >20 years was 10,428 during the survey, so the study captured more than a quarter (approximately 27 %) of the target population.

The Hakusan area, formerly Hakusan town, was merged as part of Tsu city in 2006 and is located in a semi-mountainous area. Upon survey, the proportion of people aged ≥65 years was 32.8 %, which was higher than that of Japan’s average of 24.1 %. The Hakusan area was chosen for this study because it was once an individual municipality that was fully equipped with fundamental life-related facilities, including town offices, schools, and medical care facilities.

Although the surveyed area is small, it has 83 communities ranging from small to relatively large. The study included respondents in each community. Social capital is thought to have an effect in small communities as mentioned earlier. However, in some communities, the number of respondents was <10. Social capital indicators in these communities could be imprecisely calculated. Therefore, we did not include the respondents living in 10 small communities in the analysis. As a result, 73 communities were included. The number of respondents ranged from 11 to 126 (mean: 36.8).

**Fig. 2:**
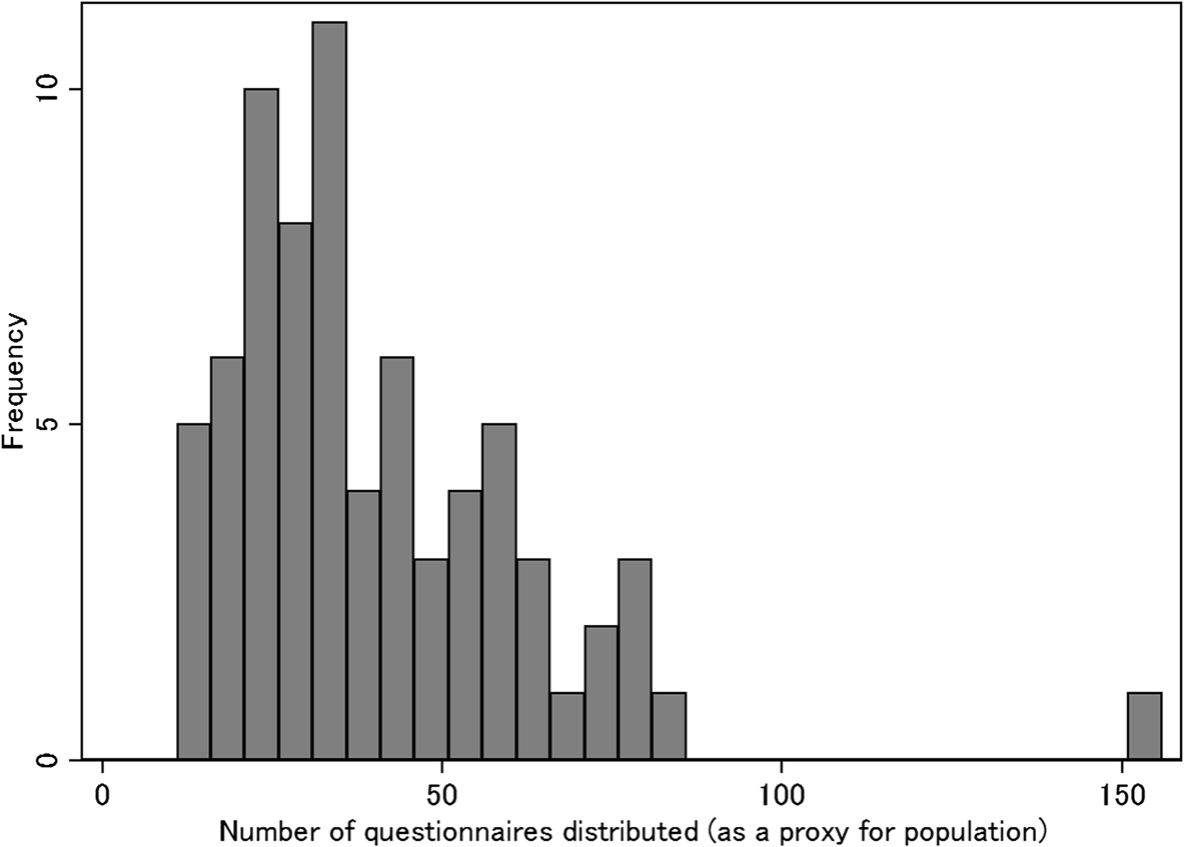
Distribution of the size of communities (73 communities)

The actual number of inhabitants in each community was not identified from the official statistics because community is not an administrative unit. In addition, the response rate of each community varied from 80 to 100 %. Therefore, we considered the number of questionnaires distributed as representative of the actual population. The average number of questionnaires distributed was 38.5 (range, 11–154). As mentioned previously, the survey captured 27 % of entire population (≥20 years) of Hakusan area. Consequently, the actual average population of each community is approximately 143 (38.5 × [100/27]) with a range of 41 (11 × [100/27])–570 (154 × [100/27]). Figure 2 shows the different community sizes and the number of questionnaires distributed.

We studied elderly respondents aged ≥60 years (*N* = 1016; males = 490; females = 526) from the entire sample. In this study, we calculated the dissimilarity index (DI) between the population of Hakusan (≥60 years) and the analytic sample [41]. DI of 0.086 indicated that the difference in sex and age between the population of Hakusan and that of the analytic sample was only 8.6 %. Therefore, we concluded that the analytic sample almost represented the entire elderly population of Hakusan.

### Refraining from medical care

Refraining from medical care was a dependent variable in the estimation. For the survey question “Have you ever refrained from visiting a doctor when medical care was needed?,” a binary outcome (1 = often or sometimes; 0 = seldom or never) was created.

### Social capital

Two main independent social capital variables, trust and reciprocity, were included. The survey included the following questions: “In general, do you think that people can be trusted?” and “In many cases, do you think that most people are willing to help others?” Answers had to be provided from one of the three following options for both questions: yes (2), depends on circumstances (1), and no (0). We assigned the values to each answer as shown in parentheses. Social capital indicators were created by calculating the mean value of the above responses within the community, but excluding each respondent’s contribution to the mean value. This community level trust and reciprocity were calculated using all individuals ≥20 years old who lived in that community.

### Community size

As mentioned earlier, we used the number of questionnaires distributed as representative of the actual population. We divided the communities into three equally sized parts according to the number of participants: small (≤39), medium (40–59), and large (≥60). This division aimed to test the inverse U shape effect of social capital by community size [40].

### Estimation strategy

We estimated two social capital entities in separate equations, because of the possible multicollinearity error (*R* = 0.532). We used a multilevel binary logit model:

$$ {Y}_{ij}=\alpha +{\beta}_1\left({X}_{ij}-{X}_j\right)+{\beta}_2{X}_j+{\beta}_3{X}_j\cdot {P}_j+{\beta}_4{P}_j+{\gamma}_1{Z}_{1,ij}+\cdots +{\gamma}_k{Z}_{k,ij}+{\mu}_i+{\varepsilon}_{ij},i=1,\dots, n,j=1,\dots, m, $$

*Y*_*ij*_ is a binary dependent variable of refraining from medical care of individual *i*, *X*_*j*_ is a social capital indicator, and *X*_*ij*_ is an answer for trust or reciprocity of individual *i* in community *j*. Individual trust and reciprocity are used as a community-mean centering variable. *P*_*j*_ is a community size and used to examine the different association between social capital and refraining from medical care by community size. *Z*_*k*,*ij*_ are control variables, *μ*_*j*_ is an unobservable community-level effect, and *ε*_*ij*_ is an idiosyncratic error. Descriptive statistics are shown in Table 1.

**Table 1:** Descriptive statistics of estimation sample

Variables	Mean	SD	Min	Max
Refraining from medical care	0.175	0.380	0	1
Trust (individual, centered)	0.013	0.484	−1.429	1.231
Trust (community)	1.163	0.089	0.769	1.778
Reciprocity (individual, centered)	0.034	0.535	−1.458	1.000
Reciprocity (community)	1.215	0.091	1.000	1.750
Community size				
Small	0.365	0.482	0	1
Medium	0.335	0.472	0	1
Large	0.300	0.459	0	1
Male	0.518	0.500	0	1
Age				
60–69	0.473	0.500	0	1
70–79	0.315	0.465	0	1
80	0.212	0.409	0	1
Education				
Elementary/junior high	0.420	0.494	0	1
High	0.428	0.495	0	1
Junior college	0.071	0.257	0	1
College/graduate	0.081	0.273	0	1
Marital status				
Married	0.744	0.437	0	1
Not married	0.029	0.167	0	1
Widowed	0.199	0.399	0	1
Divorced	0.029	0.167	0	1
Having paid work	0.380	0.486	0	1
Household annual income				
<200	0.334	0.472	0	1
200–399	0.352	0.478	0	1
400–799	0.161	0.368	0	1
≥800	0.041	0.199	0	1
No response	0.111	0.315	0	1
Number of families living with				
Zero	0.167	0.373	0	1
One	0.400	0.490	0	1
Two or three	0.313	0.464	0	1
Four or more	0.120	0.325	0	1
Years living in present place				
<10	0.069	0.253	0	1
10–19	0.062	0.241	0	1
20–29	0.060	0.238	0	1
≥30	0.809	0.393	0	1
Self-rated health	0.377	0.485	0	1
Smoking				
Now	0.133	0.340	0	1
Past, not now	0.231	0.422	0	1
Never	0.636	0.481	0	1
Drinking	0.438	0.496	0	1
Having family doctor	0.834	0.373	0	1
Car driver to hospital				
Respondents	0.618	0.486	0	1
Family members	0.345	0.476	0	1
Others	0.036	0.187	0	1
Having close neighbors	0.855	0.352	0	1
Having close friends other than neighbors	0.891	0.312	0	1

*N* = 1016, *SD* standard deviation

This equation was estimated by a random intercept model to control the unobservable community-level effect on refraining from medical care. However, the results made no difference with the results of normal logit model. Thus, we show the results of the logit model. We used individual trust or reciprocity as a community-mean centering variable. This means that trust or reciprocity at the community and individual levels were orthogonal in the analysis. Moreover, we calculated the social capital within the community, but excluding each respondent’s contribution to that. Therefore, community level trust or reciprocity was independent of individual level trust or reciprocity [42]. For statistical analysis, we used the software Stata 13 (Stata Corp, College Station, TX).

## Results

The rate of elderly respondents refraining from medical care in the Hakusan area was 17.5 % (178/1016). Furthermore, respondents who refrained from medical care were questioned regarding their reasons (*N* = 178). Answers included the following (multiple answers possible): a poor financial situation (15.2 %); difficulty in going out (21.3 %); having no reliable doctor (11.2 %); being too busy (26.4 %); the medical center being too far away (25.3 %); and others (16.9 %). These values indicate that we have to control economic conditions, time constraints, and means of transportation to determine the precise association between social capital and refraining from medical care.

**Fig. 3:**
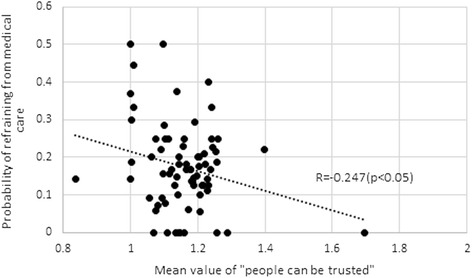
Trust and refraining from medical care (73 communities)

**Fig. 4:**
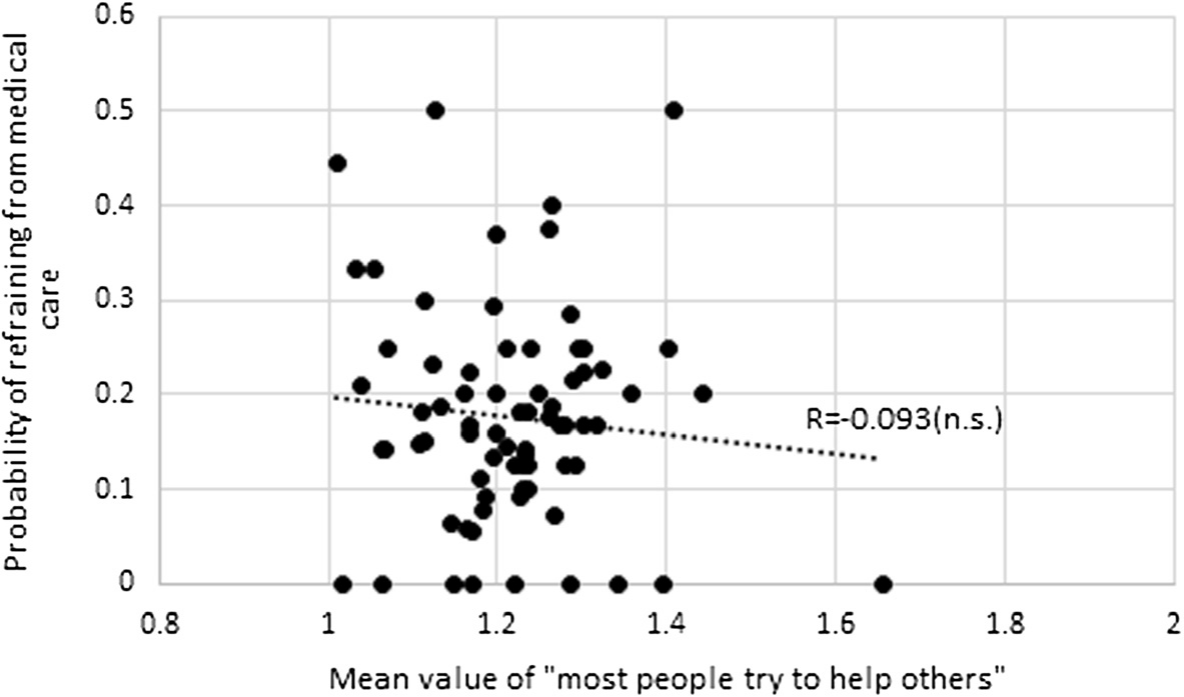
Reciprocity and refraining from medical care (73 communities). “n.s.” indicates statistically insignificant at 10 % level

Results regarding the simple associations between the community level social capital and experience rate of refraining from medical care in the community are shown in Fig. 3 (trust) and Fig. 4 (reciprocity), showing a negative correlation. Although the relation between reciprocity and refraining from medical care is statistically insignificant, the relation between trust and refraining from medical care is significant at the 5 % level. These results suggest that refraining from medical care is less likely in a community with a high level of generalized trust. However, this result may have been confounded by other factors. Therefore, we confirmed the effects of social capital using multivariate analysis.

We analyzed the association between social capital and the probability of refraining from medical care (Table 2). Since we used the interaction term for social capital, we have to calculate the marginal effects [43, 44]. The marginal effects of trust and reciprocity in communities of all sizes are shown in Table 3. Generalized trust shows the negative sign and 10 % level significance in small communities only, but was not significant in medium and large communities. In small communities, an increase of 0.1 in community level trust decreases the probability of refraining from medical care for the elderly by 4 %. Reciprocity are not significant at all. These results suggested that generalized trust and the probability of the elderly refraining from medical care had an association, but that this association was affected by the community size.

**Table 2:** Estimation results of two separate models predicting refraining from medical care

	Trust		Reciprocity	
	Coef.	SE	Coef.	SE
Trust (individual)	−0.326	0.197*		
Trust (community)	1.733	1.931		
Reciprocity (individual)			0.076	0.172
Reciprocity (community)			2.326	1.796
Community size (ref: Medium)				
Small	5.618	3.005*	4.384	2.680
Large	1.839	3.063	−0.991	4.005
Social capital # Community size				
Trust (community) # Small	−4.568	2.564*		
Trust (community) # Large	−1.531	2.616		
Reciprocity (community) # Small			−3.384	2.202
Reciprocity (community) # Large			0.787	3.285
Male	−0.195	0.248	−0.172	0.248
Age (ref: 60–69)				
70–79	−0.181	0.238	−0.204	0.238
≥80	0.141	0.316	0.089	0.315
Education (ref: High)				
Elementary/junior high	0.061	0.211	0.055	0.211
Junior college	−0.330	0.403	−0.375	0.402
College/graduate	−0.886	0.446**	−0.897	0.443**
Marital status (ref: Married)				
Not married	0.495	0.470	0.507	0.470
Widowed	−0.014	0.276	−0.029	0.274
Divorced	0.178	0.479	0.212	0.476
Having a paid work	0.522	0.221**	0.555	0.221**
Household annual income (ref: 400–799)				
<200	0.217	0.307	0.256	0.307
200–399	0.493	0.297*	0.525	0.297*
≥800	−0.104	0.547	−0.126	0.543
No response	0.342	0.377	0.408	0.377
Number of families living with (ref: Zero)				
One	−0.234	0.273	−0.275	0.271
Two	−0.270	0.287	−0.274	0.285
Three or more	0.054	0.347	0.031	0.345
Years living in present place (ref: ≥30)				
<10	0.445	0.342	0.448	0.343
10–19	0.749	0.339**	0.770	0.337**
20–29	−0.695	0.473	−0.702	0.473
Self-rated health	1.047	0.189***	1.067	0.188***
Smoking (ref: Never)				
Now	0.350	0.283	0.334	0.283
Past, not now	0.060	0.273	0.033	0.273
Drinking	−0.074	0.211	−0.109	0.210
Having family doctor	−0.727	0.236***	−0.728	0.236***
Car driver to hospital (ref: Respondents)				
Family members	0.339	0.231	0.372	0.231
Others	0.422	0.446	0.429	0.443
Having close neighbors	−0.030	0.259	−0.074	0.262
Having close friends other than neighbors	−0.521	0.283*	−0.593	0.282**
Constant	−3.554	2.321	−4.192	2.219*
Log likelihood	−421.4		−422.7	
Likelihood ratio test	100.1***		97.5***	
Pseudo R2	0.106		0.103	
Number of observations	1016			

***: *p* < 0.01, **: *p* < 0.05, *: *p* < 0.1; *Coef*. coefficient, *SE* standard error

**Table 3:** Different marginal effects of trust and reciprocity on refraining from medical care by community size

	Community size	Marginal effects	SE	*p*-value
Trust	Small	−0.398	0.233	0.088
	Medium	0.205	0.224	0.359
	Large	0.025	0.224	0.912
Reciprocity	Small	−0.152	0.189	0.421
	Medium	0.289	0.227	0.202
	Large	0.380	0.340	0.264

*SE* standard error

## Discussion

Experience rate of refraining from medical care was 17.5 % in this study. In the Aichi Prefecture, near Mie Prefecture, approximately 10 % of people aged ≥65 years refrained from medical care in the past year [15]. Our rate was higher than that reported previously; we considered three possible reasons. First, our experience rate was not limited to the past year. Second, the Mie Prefecture is more rural and thus, presents more difficulty accessing medical care than the Aichi Prefecture. Third, our sample included people aged ≥60 years and had younger subjects than the sample in Aichi. Previous studies reported younger people are more likely to refrain from medical care [13, 14, 45]. Therefore, we consider the rate of refraining from medical care (17.5 %) in our sample to be appropriate.

We found a slight association between generalized trust and refraining from medical care in small communities. As mentioned earlier, a paper by Takagi et al. investigating the association between social capital and crime in Japan found that (in both small and large communities) social capital had a significant effect on preventing crime, although there was no effect in medium-sized communities [40]. This finding was important for our study because we divided the communities of our study population into three categories according to the number of participants: small, medium, and large communities. The large communities in our sample were almost the same as the medium-sized community in the previously mentioned study by Takagi et al. on a Japanese population [40]. This proved that social capital may have an association with medical care access in a small community, but not in a large community.

Mechanisms linking social capital to elderly people refraining from medical care, at least in the small communities, are thought to be as follows. First, several elderly people selected transportation means to medical facilities as the main reason for refraining from medical care: difficulty in going out (21.3 %) and the medical center being too far away (25.3 %). In a community where residents highly trust each other, people may take elderly people by car to visit a doctor or help them when using public transportation. Second, a poor financial situation (15.2 %) caused some elderly people to refrain from medical care. In a high-level trust community, people may be more likely to financially help the elderly. This study confirmed that in Japan, one of the social capitals had a slight association with the probability of elderly people refraining from medical care. However, these mechanisms are effective only in a small community in Mie Prefecture. Moreover, we have to keep in mind that this result may be caused by omitted variable bias or reverse causality.

**Fig. 5:**
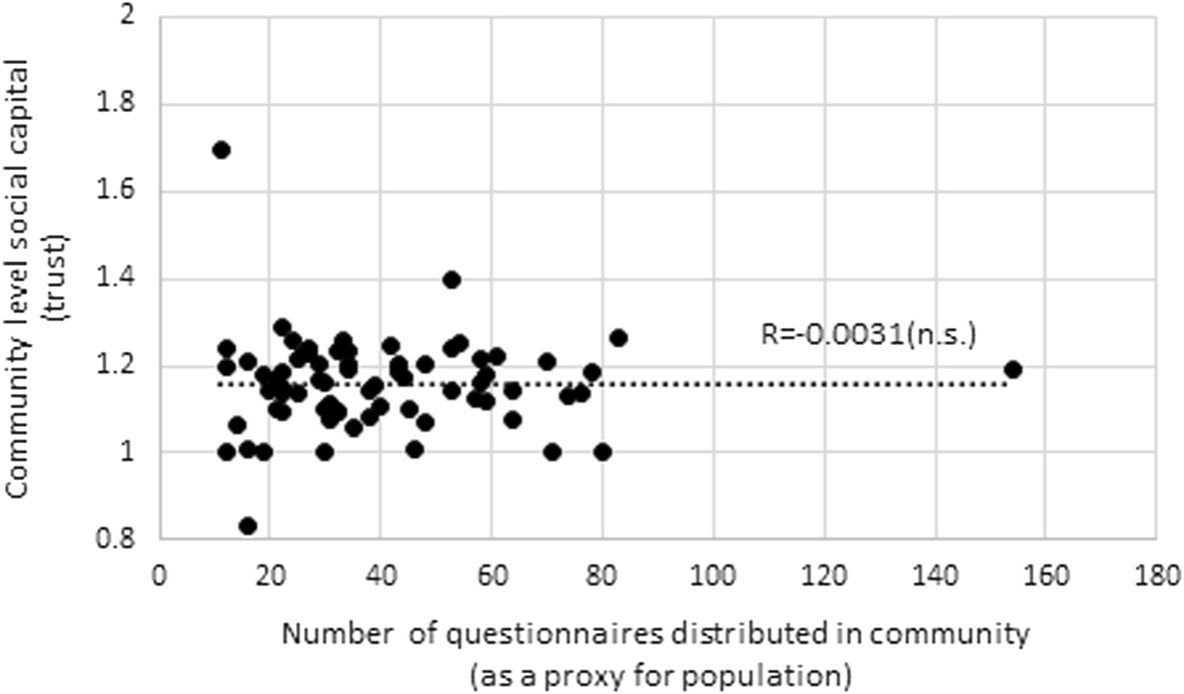
Community size and community level trust. “n.s.” indicates statistically insignificant at 10 % level

**Fig. 6:**
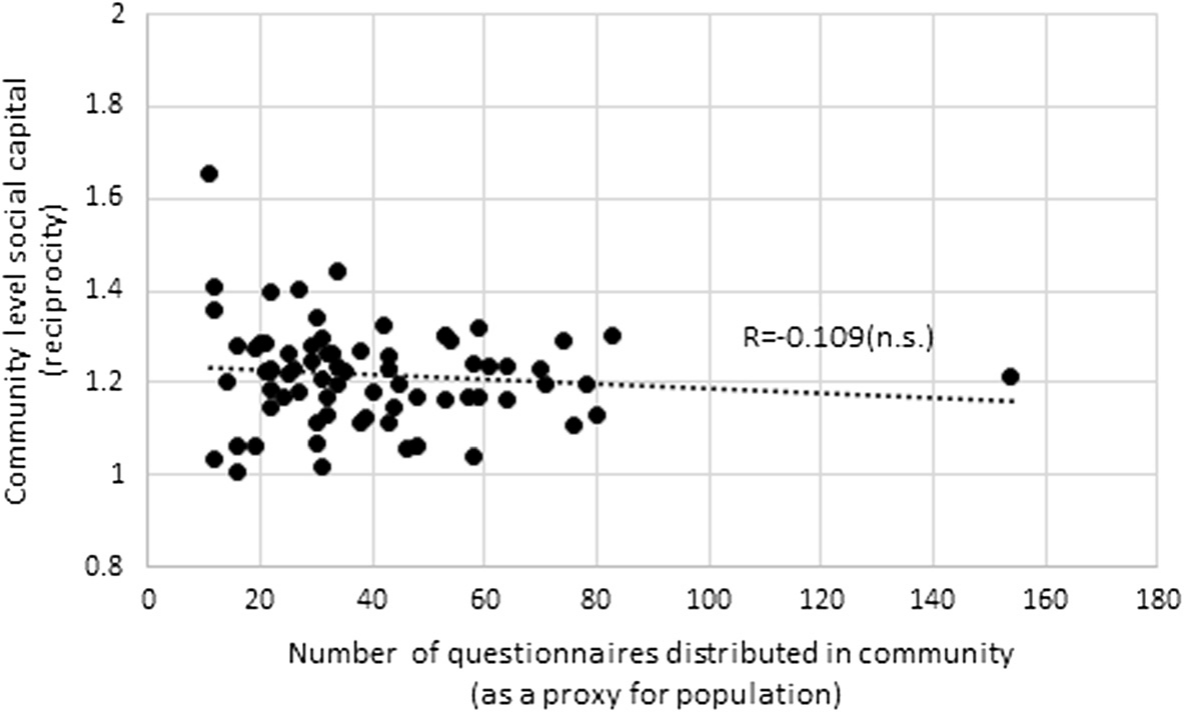
Community size and community level reciprocity. “n.s.” indicates statistically insignificant at 10 % level

Figures 5 and 6 show the correlation of community size with trust and reciprocity, respectively, with the community size having no association with social capital. Therefore, we conclude that the difference of association between social capital and refraining from medical care is caused by the size of community not by the degree of social capital. Social capital is probably ineffective for large communities because voluntary cooperation may be more difficult on a larger scale than on a smaller scale.

As mentioned in the Background section, the increase in the number of small communities is a crucial problem in Japan. Moreover, the association between social capital of the community and individual behavior was affected by community size. This paper challenged these problems by using data from a small area. However, these findings cannot be generalized directly without using national representative data [46].

## Conclusions

This study investigated the relation between community level social capital and refraining from medical care among the elderly in Japan. We found that generalized trust is slightly but negatively associated with the probability of the elderly refraining from medical care in small communities only. Many studies in other countries found a positive association between social capital in the neighborhood and medical care access [26–33]. We found a slight association in Japan. Improvement in the social capital of a community cannot occur spontaneously and requires the cooperation of public organizations [42]. Therefore, local governments have to establish high social capital in small communities or organize official groups to help the elderly.

## Abbreviation

DI, dissimilarity index

## Additional file

Additional file 1: Questionnaire. English language version of the questionnaire. (DOCX 99 kb)

## References

[1] Gornick ME, Eggers PW, Reilly TW, Mentnech RM, Fitterman LK, Kucken LE (1996). Effects of race and income on mortality and use of services among Medicare beneficiaries. N Engl J Med.

[2] Braveman PA, Egerter SA, Cubbin C, Marchi KS (2004). An approach to studying social disparities in health and health care. Am J Public Health.

[3] Westin M, Åhs A, Persson KB, Westerling R (2004). A large proportion of Swedish citizens refrain from seeking medical care—lack of confidence in the medical services a plausible explanation?. Health Policy.

[4] Åhs A, Westerling R (2006). Health care utilization among persons who are unemployed or outside the labour force. Health Policy.

[5] Westin M, Westerling R (2006). Health and healthcare utilization among single mothers and single fathers in Sweden. Scand J Public Health.

[6] Wamala S, Merlo J, Bostrom G, Hagstedt C (2007). Perceived discrimination, socioeconomic disadvantage and refraining from seeking medical treatment in Sweden. J Epidemiol Community Health.

[7] Molarius A, Simonsson B, Linden-Bostrom M, Kalander-Blomqvist M, Feldman I, Erikson HG (2014). Social inequalities in self-reported refraining from health care due to financial reasons in Sweden: health care on equal term?. BMC Health Serv Res.

[8] Health and Global Policy Institute. 2007. Public Survey on Healthcare in Japan. (in Japanese) http://www.hgpi.org/handout/2010-02-03_51_616703.pdf. Accessed 27 Nov 2014.

[9] Health and Global Policy Institute. 2008. Public Survey on Healthcare in Japan. (in Japanese) http://www.hgpi.org/handout/2009-12-14_34_275989.pdf. Accessed 27 Nov 2014.

[10] National Institute of Population and Social Security Research. The National Survey on Social Security 2007: summary of the results. (in Japanese) http://www.ipss.go.jp/ss-seikatsu/j/jittai2007/janda/jittai2007.pdf. Accessed 27 Nov 2014.

[11] Toyokawa T, Murakami K, Kaneto C, Kobayashi T (2012). Access to health care and horizontal equity. Iryo Shakai.

[12] Abe A. Who does refrain from medical care: a preliminary study using J-SHINE. CIS Discussion Paper Series. 2013; No.603. (in Japanese).

[13] Hanibuchi T (2010). Inequalities in health and health care access: analysis of access to medical care using JGSS-2008. JGSS Res Ser.

[14] Murata C (2010). Concern about health care and socio-economic status: analysis using JGSS-2008 data. JGSS Res Ser.

[15] Murata C, Yamada T, Chen C, Ojima T, Hirai H, Kondo K (2010). Barriers to health care among the elderly in Japan. Int J Environ Res Public Health.

[16] National Institute of Population and Social Security Research. Regional Population Projection for Japan 2010–2040. Population Research Series. 2013;330. (in Japanese)

[17] Ministry of Internal Affairs and Communications. Securing Medical Care in Depopulated Area 2001. http://www.soumu.go.jp/main_sosiki/jichi_gyousei/c-gyousei/2001/kaso/pdf/kasokon20_05_02_s3.pdf. Accessed 21 Dec 2015. (in Japanese)

[18] Hirai H, Kondo K (2009). Factors associated with participation in health examination among the elderly population: comparison of the three regional characteristics. J Rural Plann Assoc.

[19] Putnam RD (1995). Bowling alone: America’s declining social capital. J Democr.

[20] Bourdieu P, Richardson J (1986). The forms of capital. Handbook of theory and research for the sociology of education.

[21] Kawachi I, Takao S, Subramanian SV, Kawachi I, Takao S, Subramanian SV (2013). Introduction. Global perspective on social capital and health.

[22] Takagi D, Kawachi I, Takao S, Subramanian SV (2013). Neighborhood social capital and crime. Global Perspective on Social Capital and Health.

[23] Derose KP, Varda DM (2009). Social capital and health care access: a systematic review. Med Care Res Rev.

[24] Kawachi I, Berkman LF, Berkman LF, Kawachi I, Glymour MM (2014). Social capital, social cohesion, and health. Social epidemiology.

[25] Kawachi I, Kennedy BP, Glass R (1999). Social capital and self-rated health: a contextual analysis. Am J Public Health.

[26] Veenstra G (1999). Social capital, SES and health: an individual-level analysis. Soc Sci Med.

[27] Veenstra G (2002). Social capital and health (plus wealth, income inequality and regional health governance). Soc Sci Med.

[28] Rose R (2000). How much does social capital add to individual health?. Soc Sci Med.

[29] Kawachi I, Berkman L, Kawachi I, Berkman L (2000). Social cohesion, social capital, and health. Social epidemiology.

[30] Subramanian SV, Kim DJ, Kawachi I (2002). Social trust and self-rated health in US communities: a multilevel analysis. J Urban Health.

[31] Kim DJ, Kawachi I (2006). A multilevel analysis of key forms of community- and individual-level social capital as predictors of self-rated health in the United States. J Urban Health.

[32] Ichida Y, Kondo K, Hirai H, Hanibuchi T, Yoshikawa G, Murata C (2009). Social capital, income inequality and self-rated health in Chita peninsula, Japan: a multilevel analysis of older people in 25 communities. Soc Sci Med.

[33] Ahren MM, Hendryx MS (2003). Social capital and trust in providers. Soc Sci Med.

[34] Drukker M, Driessen G, Krabbendam L, van Os J (2004). The wider social environment and mental health service use. Acta Psychiatr Scand.

[35] Greenberg GA, Rosenheck RA (2003). Managerial and environmental factors in the continuity of mental health care across institutions. Psychiatr Serv.

[36] Hendryx MS, Ahren MM, Lovrich NP, NcCurdy AH (2002). Access to health care and community social capital. Health Serv Res.

[37] Prentice JC (2006). Neighborhood effects on primary care access in Los Angeles. Soc Sci Med.

[38] van der Linden J, Drukker M, Gunther N, Feron F, van Os J (2003). Children’s mental health service use, neighborhood socioeconomic deprivation, and social capital. Soc Psychiatry Psychiatr Epidemiol.

[39] Chaix B, Merlo J, Subramanian SV, Lynch J, Chauvin P (2005). Comparison of a spatial perspective with the multilevel analytical approach in neighborhood studies: the case of mental and behavioral disorders due to psychoactive substance use in Malmö, Sweden, 2001. Am J Epidemiol.

[40] Takagi D, Ikeda K, Harihara M, Kobayashi T (2011). Does crime control effect of social capital vary depending on the range of “neighborhood”? Analysis using GIS and physical distances between residents. Theory Appl GIS.

[41] Duncan OD, Duncan B (1955). Residential distribution and occupational stratification. Am J Sociol.

[42] Kawachi I, Subramanian SV, Kim D, Kawachi I, Subramanian SV, Kim D (2008). Social capital and health: a decade of progress and beyond. Social capital and health.

[43] Ai C, Norton EC (2003). Interaction terms in logit and probit models. Econ Lett.

[44] Karaca-Mandic P, Norton EC, Dowd B (2012). Interaction terms in nonlinear models. Health Serv Res.

[45] Perry M, Williams RL, Wallerstein N, Waitzkin H (2008). Social capital and health care experiences among low-income individuals. Am J Public Health.

[46] Doorslaer EV, Masseria C, Koolman X (2006). Inequalities in access to medical care by income in developed countries. CMAJ.

