# Supplementary material for: Social capital and refraining from medical care among elderly people in Japan
Source: BMC Health Serv Res. 2016 Aug 2;16:331. doi: 10.1186/s12913-016-1599-8 (PMC4970323; doi:10.1186/s12913-016-1599-8)
Supplement: Additional file 1: — Questionnaire. English language version of the questionnaire. (DOCX 99 kb) [file 12913_2016_1599_MOESM1_ESM.docx]

Survey on medical care, health, and welfare in the Hakusan area

Faculty of Medicine, Mie University

Faculty of Humanities, Law, and Economics, Mie University

***1. About you and your family***

【1-1】 What is your sex? Choose one that applies. How old are you?

1. Male 2. Female _________ years old

【1-2】 Where do you live? Please state the name of the local self-government body.

Name of the local self-government body _____________________________

【1-3】 How long have you lived at your current address? Choose one that applies.

1. Less than 5 years 2. 5-9 years 3. 10-19 years

4. 20-29 years 5. 30 years or more

【1-4】 What is the highest level of school you attended? Choose one that applies.

1. Elementary / junior high school (higher elementary school in the old system)

2. High school (Junior high school in the old system)

3. College of technology / 2-year college

4. University / graduate school

【1-5】 Where is your workplace / school? Choose one that applies. (If you are both working and attending school, choose your workplace.)

1. Not attending / at home 2. Hakusan area 3. Ichishi area 4. Hisai area

5. Misato area 6. Misugi area 7. Other area in Tsu city 8. Matsuzaka city

9. Iga city 10. Nabari city 11. Other city / town (____________________)

12. Outside Mie prefecture

【1-6】 Are you employed or engaged in a different activity? If you are employed, is it your primary activity? Choose one that applies.

**If you are employed:** **If you are not employed:**

1. Primary activity is work 5. I am a housekeeper

2. Primary activity is housekeeping 6. I am a student

3. Primary activity is attending school 7. I am unemployed

4. Primary activity is something else 8. Other

【1-7】 Which one of the following categories best describe your job? Choose one that applies.

1. Full-time 2. Part-time 3. Dispatched 4. Contract 5. Entrusted

6. Executive 7. Self-employed 8. Family worker 9. Doing piece work at home

10. Other (_____________________)

【1-8】 What kind of job are you engaged in?

1. Administrative / managerial 2. Professional / engineering

3. Clerical 4. Sales

5. Service 6. Security

7. Agriculture / forestry / fishery 8. Manufacturing process

9. Transport / machine operation 10. Construction / mining

11. Carrying / cleaning / packaging / etc. 12. Unclassified

【1-9】 How many hours do you usually work per week? Choose one that applies.

1. Less than 15 hours 2. 15-19 hours 3. 20-21 hours 4. 22-29 hours

5. 30-34 hours 6. 35-42 hours 7. 43-45 hours 8. 46-48 hours

9. 49-59 hours 10. 60-64 hours 11. 65 hours or more

【1-10】 What is your annual household income? Choose one that applies.

1. Less than 1 million yen 2. 1-2 million yen 3. 2-3 million yen

4. 3-4 million yen 5. 4-5 million yen 6. 5-6 million yen

7. 6-7 million yen 8. 7-8 million yen 9. 8-9 million yen

10. 9-10 million yen 11. 10 million yen or over

【1-11】 What are your sources of financial support? Check all that apply.

1. Labor income 2. Self-employment income 3. Asset income

4. National pension 5. Employee pension 6. Seamen’s insurance

7. Mutual aid pension 8. Pension 9. Private pension (company/personal pension)

10. Unemployment benefits 11. Social welfare benefits 12. Savings

13. Remittance 14. Other (________________)

【1-12】 Are you married? Choose one that applies.

1. Married 2. Not married 3. Widowed 4. Divorced

【1-13】 Do you have children? Choose one that applies.

1. Yes 2. No

【1-14】 Do you living with anyone? Choose one that applies.

1. Yes 2. No

【1-15】 Who do you live with? Please answer one by one. If you are living alone, you don’t have to answer the following. If you live with more than 6 individuals, please add them in the blank space.

|  | Relationship to you | | | | | | | | | | | Age | Sex | Does this person earn an income? |
| --- | --- | --- | --- | --- | --- | --- | --- | --- | --- | --- | --- | --- | --- | --- |
|  | Spouse | Child | Son/daughter-in-law | Father | Mother | Father-in-law | Mother-in-law | Grandchild | Sibling | Other relative | Other |  |  |  |
| (1) | 1 | 2 | 3 | 4 | 5 | 6 | 7 | 8 | 9 | 10 | 11 |  | M / F | Yes / No |
| (2) | 1 | 2 | 3 | 4 | 5 | 6 | 7 | 8 | 9 | 10 | 11 |  | M / F | Yes / No |
| (3) | 1 | 2 | 3 | 4 | 5 | 6 | 7 | 8 | 9 | 10 | 11 |  | M / F | Yes / No |
| (4) | 1 | 2 | 3 | 4 | 5 | 6 | 7 | 8 | 9 | 10 | 11 |  | M / F | Yes / No |
| (5) | 1 | 2 | 3 | 4 | 5 | 6 | 7 | 8 | 9 | 10 | 11 |  | M / F | Yes / No |
| (6) | 1 | 2 | 3 | 4 | 5 | 6 | 7 | 8 | 9 | 10 | 11 |  | M / F | Yes / No |

【1-16】 Please answer the following questions about your family members (including family members not living with you).

| 1. When problems arise, family members discuss and make decisions together. | Yes / No |
| --- | --- |
| 2. Each family member’s role in the family is clear, but sometimes they complement each other. | Yes / No |
| 3. When problems arise, someone makes a decision arbitrarily. | Yes / No |
| 4. Family members can change their roles in the family freely. | Yes / No |
| 5. Family members abide by the rules of the family. | Yes / No |
| 6. Family members have never followed through on promises. | Yes / No |
| 7. When problems arise, family members discuss, but a specific person makes a final decision. | Yes / No |
| 8. Family members have never followed through on decisions. | Yes / No |
| 9. Family members generally enjoy their time separately, but sometimes spend it together. | Yes / No |
| 10. When children get depressed, parents worry about them, but do not ask about the reason. | Yes / No |
| 11. If I have troubles, I sometimes consult my family members. | Yes / No |
| 12. Family members often have physical contact with each other. | Yes / No |
| 13. Family members have no relationship except for business. | Yes / No |
| 14. Family members have only the minimum conversation necessary. | Yes / No |
| 15. Family members sometimes spend holidays together, and sometimes go out with friends. | Yes / No |
| 16. When someone returns home late, family members sit up waiting for their return. | Yes / No |

***2. About your feelings***

【2-1】 Please indicate how accurate the following statements are for describing your usual feelings.

|  | Almost never | Sometimes | Often | Almost always |
| --- | --- | --- | --- | --- |
| 1. I feel pleasant |  |  |  |  |
| 2. I tire quickly |  |  |  |  |
| 3. I feel like crying |  |  |  |  |
| 4. I wish I could be as happy as others seem to be |  |  |  |  |
| 5. I am losing out on things because I can’t make up mind soon enough |  |  |  |  |
| 6. I felt rested |  |  |  |  |
| 7. I am calm, cool and collected |  |  |  |  |
| 8. I feel that difficulties are piling up so that I can not overcome them |  |  |  |  |
| 9. I worry too much over something that really doesn’t matter |  |  |  |  |
| 10. I am happy |  |  |  |  |
| 11. I am inclined to take things hard |  |  |  |  |
| 12. I lack self-confidence |  |  |  |  |
| 13. I feel secure |  |  |  |  |
| 14. I try to avoid facing crises or difficulty |  |  |  |  |
| 15. I feel blue |  |  |  |  |
| 16. I am content |  |  |  |  |
| 17. Some unimportant thoughts runs through my mind and bothers me |  |  |  |  |
| 18. I take disappointments so keenly that I can’t put them out of my mind |  |  |  |  |
| 19. I am a steady person |  |  |  |  |
| 20. I get in a state of tension or turmoil as I think over my recent concerns and interests |  |  |  |  |

***3. About your health***

【3-1】 Overall, how would you rate your health during the past 4 weeks? Choose one that applies.

1. Excellent 2. Very good 3. Good

4. Fair 5. Poor 6. Very poor

【3-2】 During the past 4 weeks, how much did physical health problems limit your usual physical activities (such as walking or climbing stairs)? Choose one that applies.

1. Not at all 2. Very little 3. Somewhat 4. Quite a lot

5. Could not do physical activities

【3-3】 During the past 4 weeks, how much difficulty did you have doing your daily work, both at home and away from home, because of physical health? Choose one that applies.

1. None at all 2. A little bit 3. Some 4. Quite a lot

5. Could not do daily work

【3-4】 How much bodily pain have you had during the past 4 weeks? Choose one that applies.

1. None 2. Very mild 3. Mild

4. Moderate 5. Severe 6. Very severe

【3-5】 During the past 4 weeks, how much energy did you have? Choose one that applies.

1. Very much 2. Quite a lot 3. Some 4. A little 5. None

【3-6】 During the past 4 weeks, how much did your physical health or emotional problems limit your usual social activities with family or friends? Choose one that applies.

1. Not at all 2. Very little 3. Somewhat 4. Quite a lot

5. Could not do social activities

【3-7】 During the past 4 weeks, how much have you been bothered by emotional problems (such as feeling anxious, depressed, or irritable)? Choose one that applies.

1. Not at all 2. Slightly 3. Moderately 4. Quite a lot 5. Extremely

【3-8】 During the past 4 weeks, how much did personal or emotional problems keep you from doing your usual work, school, or other daily activities? Choose one that applies.

1. Not at all 2. Very little 3. Somewhat 4. Quite a lot

5. Could not do daily activities

【3-9】 Have you refrained from going out due to fear of falling? Choose one that applies.

1. Yes 2. No

【3-10】 Are you able to see well enough to read (using glasses if needed)? Choose one that applies.

1. Yes (I can read books easily) 2. No (Not clear / almost unable)

【3-11】 Are you able to chew all kinds of food (using artificial teeth if needed)? Choose one that applies.

1. Yes, I’m able to chew almost all kinds of food

2. No, I’m not able to chew many kinds of food

***4. About smoking and drinking***

【4-1】 Do you smoke? Choose one that applies.

1. I am a smoker 2. I used to smoke, but I have stopped smoking 3. I have never smoked

【4-2】 How many cigarettes do you smoke / did you smoke per day?

________________ cigarettes per day

【4-3】 How many years have you smoked? If you stopped smoking for some years, please indicate the total number of years you were a smoker.

________________ year(s)

【4-4】 Do you drink alcoholic drinks? Choose one that applies. If yes, at what age did you start to drink alcohol?

1. Yes (____________ years old) 2. No

【4-5】 Have you ever felt that you should decrease your amount of drinking? Choose one that applies.

1. Yes 2. No

【4-6】 Have you been bothered when someone disapproved of your drinking? Choose one that applies.

1. Yes 2. No

【4-7】 Have you felt regretful or bad about your drinking? Choose one that applies.

1. Yes 2. No

【4-8】 Have you ever drunk alcohol in the morning to calm tension or cure a hangover? Choose one that applies.

1. Yes 2. No

【4-9】How often do you drink alcoholic drinks? Choose one that applies.

1. Never 2. Once a month 3. Several times a month 4. Several times a week

5. Almost daily

【4-10】 How many drinks (see the guide for equivalents) do you usually have at a time? Choose one that applies.

**Guide:**

One go of Japanese sake = 2

Glass of wine = 1.5

Large bottle of beer = 2.5

Small cup of plum wine = 1

Double whiskey and water = 2

Glass of Japanese spirit with hot water = 1

1. 1-2 2. 3-4 3. 5-6

4. 7-9 5. 10 or more

【4-11】 How often do you have more than 5 drinks (as defined above) at a time? Choose one that applies.

1. Never 2. Less than once a month 3. Once a month

4. Once a week 5. Daily or almost daily

***5. About medical care in your community***

【5-1】 Do you have a family / home doctor? Choose one that applies. (A family / home doctor is the doctor who usually examines you, and refers you other doctors, hospitals, or related facilities if necessary.)

1. Yes 2. No

【5-2】 What is your family / home doctor’s medical facility?

Name of the medical facility ___________________________

【5-3】 How many years have you had the family/home doctor? Choose one that applies.

1. Less than 5 years 2. 5-9 years 3. 10-14 years 4. 15-19 years

5. 20-24 years 6. 25-29 years 7. 30 years or more

【5-4】 How often do you visit the family / home doctor’s medical facility? Choose one that applies.

1. Almost daily 2. 2-5 times a week 3. Once a week 4. Several times a month

5. Once a month 6. Less than once every 2 months

【5-5】 How do you travel to the medical facility? Choose one that applies.

1. Home visit 2. On foot 3. Bicycle 4. Motorcycle 5. Car (you drive)

6. Car (someone else drives) 7. Pickup car from the medical facility

8. Bus 9. Taxi 10. Train

【5-6】 How long does it take to travel to the medical facility? Choose one that applies.

1. Home visit 2. Less than 5 minutes 3. 5-14 minutes 4. 15-29 minutes

5. 30 minutes - 1 hour 6. More than 1 hour

【5-7】 How many medical facilities do you currently visit regularly (including the above medical facility)? Choose one that applies.

1. None 2. 1 facility 3. 2 facilities 4. 3 facilities 5. 4 or more

【5-8】 How often do you visit these medical facilities? Choose one that applies.

1. Almost daily 2. 2-5 times a week 3. Once a week 4. Several times a month

5. Once a month 6. Less than once every 2 months

【5-9】 How many times have you been admitted to the hospital? Choose one that applies.

1. Never 2. Once 3. 2 times 4. 3 times

5. 4 times 6. 5 times or more

【5-10】 Which of the following medical facilities do you visit when necessary? Choose one that applies.

**If you have a family / home doctor:** **If you do not have a family / home doctor:**

1 Family / home doctor’s medical facility 3 Clinic

2 Other medical facility, depending on the 4 Hospital

type of illness 5 University hospital

Name of the medical facility_________________________

【5-11】 Have you ever refrained from visiting a doctor when medical care was needed? Choose one that applies.

1. Often 2. Sometimes 3. Seldom 4. Never

【5-12】 What are your reasons for not seeking medical care? Choose all that apply.

1. Poor financial situation 2. Difficulty going out 3. No reliable doctor / medical facility

4. Busy 5. Medical facility too far away

6. Other (_______________________)

【5-13】 In the past 3 years, have you sought emergency medical care from any of the following? Please answer for each item.

| Ambulance | Yes / No |
| --- | --- |
| Tsu city pediatric emergency clinic (Mie Hospital) | Yes / No |
| Tsu city emergency clinic (Tsu City Region Plaza) | Yes / No |
| Hisai emergency clinic (Hisai-Ichishi Area Doctor Center) | Yes / No |
| Tsu city emergency and health consultation telephone service | Yes / No |
| Matsuzaka city emergency clinic (Matsuzaka City Health Center) | Yes / No |
| Matsuzaka city dental emergency clinic (Matsuzaka City Dental Center) | Yes / No |
| Emergency medical treatment at other hospitals or clinics | Yes / No |

【5-14】 In the past 5 years, how often have you received a health examination, cancer screening, or clinical survey? Choose one that applies.

1. Every year 2. Irregularly 3. Never

【5-15】 Does your household have a car or motorcycle to take to the medical facility? Choose one that applies.

1. Yes 2. No

【5-16】 When going to medical facility by car, who drives? Choose one that applies.

1. Me 2. Family member 3. Other

【5-17】 Are you anxious about not receiving adequate medical care when needed in the Hakusan area? Choose one that applies.

1. Yes 2. No

【5-18】 How satisfied are you with the medical care, health, and welfare services in Tsu city?

Very dissatisfied Very satisfied

1 2 3 4 5 6 7 8 9 10

***6. About human relations and social activities in your community***

【6-1】 Do you usually stay at home rather than going out? Choose one that applies.

1. Yes 2. No

【6-2】 How often do you usually go out for work, shopping, or walks? Choose one that applies.

1. More than once every a few days 2. Less than once a week

【6-3】 Do you have close neighbors? Choose one that applies.

1. Yes 2. No

【6-4】 Do you have close friends other than neighbors? Choose one that applies.

1. Yes 2. No

【6-5】 Do you have a hobby? Choose one that applies.

1. Yes 2. No

【6-6】 In general, do you think that people can be trusted? Choose one that applies.

1. Yes 2. Depends on circumstances 3. No

【6-7】 In many cases, do you think that most people are willing to help others? Choose one that applies.

1. Yes 2. Depends on circumstances 3. No

【6-8】 Do you have family members or relatives that do not live with you, but help you and your family members when necessary? If yes, where do they live? Choose one that applies.

1. Hakusan area 2. Tsu city 3. Mie prefecture 4. Outside Mie prefecture

5. There is no one aside from live-in family members

【6-9】 Do you participate in the following activities? Please answer for each activity.

If you choose “Yes” for “(1) Participation,” please choose the most accurate option for “(2) Organization.”

For “(3) Importance” and “(4) Future,” please choose an applicable option regardless of whether you participate.

|  | (1) Participation | (2) Organization | | | | | (3) Importance | | | (4) Future | | |
| --- | --- | --- | --- | --- | --- | --- | --- | --- | --- | --- | --- | --- |
|  |  | School | Local self-government body | Social welfare council/social worker | NPO | Other | Important | Don’t know | Unimportant | I want to continue | Don’t know | I want to quit |
| Community events | Yes / No | 1 | 2 | 3 | 4 | 5 | 1 | 2 | 3 | 1 | 2 | 3 |
| Child care support and education | Yes / No | 1 | 2 | 3 | 4 | 5 | 1 | 2 | 3 | 1 | 2 | 3 |
| Elder care support | Yes / No | 1 | 2 | 3 | 4 | 5 | 1 | 2 | 3 | 1 | 2 | 3 |
| Other volunteer activities | Yes / No | 1 | 2 | 3 | 4 | 5 | 1 | 2 | 3 | 1 | 2 | 3 |
| Health care and preventive care | Yes / No | 1 | 2 | 3 | 4 | 5 | 1 | 2 | 3 | 1 | 2 | 3 |

【6-10】 Do you know the social workers managing your community? Choose one that applies.

1. I know their names and faces 2. I know their names or faces

3. I don’t know 4. I don’t know what a social worker is

【6-11】 Are public information papers distributed to your house? Choose one that applies.

1. Yes 2. Yes, but I don’t read them 3. No 4. I don’t know

【6-12】 Can you expect support from the following people when you or your family members have health problems? Please answer for each.

| Members of the local self-government body | 1. Yes 2. No 3. Don’t know |
| --- | --- |
| Social workers | 1. Yes 2. No 3. Don’t know |
| Professionals of the social welfare council or medical facility | 1. Yes 2. No 3. Don’t know |
| Officials of the local government | 1. Yes 2. No 3. Don’t know |

【6-13】 What is the limit of the range within which people can help each other? Choose one that applies.

1. Family members and relatives 2. Local self-government body

3. Elementary school district 4. Junior high school district

5. Hakusan area 6. Tsu city

***7. About nursing care***

【7-1】 Do you currently require nursing care or care for a chronic disease / disorder? Choose one that applies.

1. Yes 2. No

【7-2】 Who provides nursing care for you? Please choose all that apply.

1 Family members or relatives

2. I use Long-Term Care Insurance service at home

3. I go to a care center for Long-Term Care Insurance service

4. I moved into a care center and use Long-Term Care Insurance service

5. I don’t use any service

6. Other (___________________)

【7-3】 Do you have family members who need nursing care or care for a chronic disease / disorder? (Please include family members who do not live with you.)

1. Yes 2. No

【7-4】 Do you care for these family members? Choose one that applies.

1. Yes 2. No

【7-5】 How many hours per day do you care for your family members? Choose one that applies. (If you provide care once every few days, choose “5. Other”)

1. Almost all day 2. Half a day 3. 2-3 hours 4. As necessary 5. Other

***8. About daily life satisfaction, etc.***

【8-1】 Who do you think should be responsible for the following? Choose a number from 1 to 5 for each.

Individuals and families Governments

| Livelihood of the elderly, handicapped, and unemployed | 1 2 3 4 5 |
| --- | --- |
| Medical care | 1 2 3 4 5 |
| Nursing care | 1 2 3 4 5 |
| Education of children | 1 2 3 4 5 |
| Raising and caring for children | 1 2 3 4 5 |

【8-2】 Overall, how satisfied are you with daily life in your community? Choose one that applies.

Very dissatisfied Very satisfied

1 2 3 4 5 6 7 8 9 10

【8-3】 Please feel free to write any concerns or frustrations you have concerning your family members’ health, or your opinions about medical care, health, and welfare in your community.

Thank you for your cooperation.
